# Exploring the Relationship between Fundamental Movement Skills and Health-Related Fitness among First and Second Graders in Korea: Implications for Healthy Childhood Development

**DOI:** 10.3390/healthcare12161629

**Published:** 2024-08-16

**Authors:** Se-Won Park, Sung-Ho Yoon, Seung-Man Lee

**Affiliations:** 1 Department of Elementary Education, Korea National University of Education, Cheongju 28173, Republic of Korea; parksewon@knue.ac.kr; 2 Department of Physical Education, Korea National University of Education, Cheongju 28173, Republic of Korea; 3 Department of Sports Science, Hankyong National University, Anseong 17579, Republic of Korea

**Keywords:** fundamental movement skills, health-related fitness, elementary school physical education, childhood, health

## Abstract

This study investigated the relationship between fundamental movement skills (FMSs) and health-related fitness (HRF) among first and second graders in South Korean elementary schools. It aimed to provide foundational data for developing physical education programs tailored to the motor development stages and fitness levels of younger elementary school students. This study utilized secondary data from the physical activity competence evaluation conducted by the Health Physical Activity Institute (HPAI). In October 2023, the HPAI evaluated the fundamental movement skills (jumping, running, hopping, static balance, dynamic balance, overhand throwing, and kicking) and health-related fitness (muscular strength, cardiorespiratory endurance, and flexibility) of 291 first and second-grade students. The collected data were analyzed through frequency and multiple regression analyses performed using SPSS software. The results revealed that higher scores in jumping and hopping are associated with greater muscular strength, cardiorespiratory endurance, and flexibility. Running had no significant effect on HRF elements. Higher scores in static balance (i.e., that used in single-leg stance) were associated with increased muscular strength, cardiorespiratory endurance, and flexibility, but dynamic balance (balance beam walking) did not have a significant effect. Higher scores in overhand throwing were associated with greater muscular strength and cardiorespiratory endurance, but kicking did not show a significant association. Overall, these findings emphasize the importance of prioritizing jumping and static balance in physical education for the well-rounded health development of first and second graders. Based on the results derived from this study, it is expected to serve as a theoretical basis for including “jumping” and “static balance” in the first and second grade curriculum of elementary schools, thereby providing essential guidance.

## 1. Introduction

The COVID-19 pandemic has made sedentary lifestyles the new normal among students, emphasizing the importance of school physical education [1,2]. Physical education, particularly in the early years of elementary school, is of vital importance due to its influence on health and growth, emotional stability and maturity, motor skill development, and the formation of basic life habits [3]. However, for first and second graders in Korea, physical education is combined with music and art as an integrated subject, resulting in insufficient continuity of physical education from kindergarten to school [4,5]. Physical education is not taught as an integrated subject in other countries [6,7,8], and even among the integrated subjects, physical education is often neglected by homeroom teachers due to the burden of managing students [9,10]. To address this issue, the National Education Commission decided in April 2024 to start physical education as a separate subject for first and second graders. This has necessitated the selection of content for physical education curricula for first and second graders in Korea.

Elementary school physical education attaches great importance to the acquisition of fundamental movement skills (FMSs). FMSs are skills that are necessary for participating in physical activities and serve as the building blocks for learning and developing more complex sports and movement skills [11]. These skills can be divided into three groups: locomotor movement skills (skills involving moving one’s body across space, e.g., running, hopping, and jumping), non-locomotor movement skills (skills focusing on body control, e.g., balancing), and manipulative skills (skills involving controlling and moving objects, e.g., overhand throwing, kicking, dribbling, and striking) [12].

The first and second grades represent a crucial stage in children’s motor development, as they lay the foundation for sports socialization and FMS acquisition [13]. It is imperative to note that FMSs are not acquired naturally because of growth and maturation [14,15]. Skills that children can acquire without adult guidance are often limited in achievement level. Insufficient learning of FMSs in childhood can lead to underdeveloped FMSs in adulthood [16]. Furthermore, insufficient mastery of FMSs can significantly impede the motor development stage, i.e., the acquisition of sports skills [17]. Therefore, to facilitate lifelong engagement in sports, opportunities must be provided from early elementary school years for the acquisition of FMSs [18]. The revised 2022 Korean Physical Education Curriculum [19] places a strong emphasis on FMSs, highlighting the need for their inclusion as a core component of physical education curricula for first and second graders.

Reduced physical activity owing to the COVID-19 pandemic has decreased students’ fitness levels. This has necessitated teaching fitness-related content in the early years of elementary school [20]. Physical activity forms the basis for maintaining and improving one’s health, and fitness is an important indicator of the health of school-age children and adolescents [21]. Fitness is broadly categorized as health-related fitness (HRF) or skill-related fitness (SRF). HRF is necessary for performing physical activities and encapsulates cardiorespiratory endurance, muscular strength, muscular endurance, body composition, and flexibility. Meanwhile, SRF is necessary for sports performance and comprises agility, balance, coordination, power, speed, and reaction time [22].

Children in lower elementary grades engage in less physical activity than those in upper elementary grades, and this phenomenon is more prominent among girls than among boys [23]. Therefore, there must be a variety of physical activity programs for students, including those in lower elementary grades, to improve their physical activity and fitness levels [24,25,26]. However, to enhance students’ fitness levels, increasing physical activity is not enough. Rather, it is important to teach fitness-related content [27]. An approach that prioritizes improving HRF while teaching the basic elements of SRF is recommended for elementary school physical education [12,28]. Additionally, aligning with the plan to expand the application of the Physical Activity Promotion System (PAPS) from grades 5–6 to grades 1–6 [29], content that enhances basic fitness must be incorporated into the curricula for lower elementary grades.

Therefore, content covering FMSs and HRF must be included in the physical education curricula of lower elementary grades. Previous studies on elementary students’ FMSs and HRF have shown a strong correlation between FMSs and HRF [30,31,32], indicating that integrated teaching of these elements is more effective [33]. However, many of these studies [30,34,35] have not fully explained the relationship between FMSs and HRF in the context of selecting content for first and second graders’ physical education curricula. This lack of clarity leads to conceptual misunderstandings and content overlap in teaching practice, impeding systematic teaching and learning.

This study clarified the relationship between FMSs and HRF among first and second graders. More specifically, this study addressed three research questions: (1) How do locomotor movement skills (running, jumping, and hopping) affect HRF (muscular strength, cardiorespiratory endurance, and flexibility)? (2) How do stability (or non-locomotor movement) skills (static and dynamic balance) affect HRF (muscular strength, cardiorespiratory endurance, and flexibility)? (3) How do manipulative skills (overhand throwing and kicking) affect HRF (muscular strength, cardiorespiratory endurance, and flexibility)? With this objective, this study aimed to provide foundational data for the development of physical education curricula for lower elementary grades. The results of this study are expected to summarize the relationship between FMS and HRF in lower elementary school grades, thereby identifying common denominators in the stages of motor and fitness development and providing foundational data for the development of systematic educational programs.

## 2. Methods

### 2.1. Participants

This study utilized secondary data from the physical activity competence evaluation conducted by the Health Physical Activity Institute (HPAI). In October 2023, the HPAI selected 291 first and second graders from elementary schools in City C, Korea, as study subjects using convenience sampling. Table 1 shows sex- and grade-based distributions of the participants. As for the gender composition of the participants, 142 male students (48.8%) participated, and 149 female students (51.2%) participated. Some 137 participants (47.1%) were aged seven, and 154 participants (52.9%) were aged eight.

### 2.2. Measurements

This study used the Physical Activity Competency Assessment [36] and the PAPS [29] to assess FMSs and HRF. In this study, the independent variables are movement skills, and the dependent variables are jumping, running, and hopping.

#### 2.2.1. FMSs

Jumping was assessed based on “jumping for distance” performed in the following manner. The participant stood with their feet shoulder-width apart and jumped forward with both feet landing on both feet. Then, the distance from the take-off point to the heel of the landing point was measured in centimeters. Finally, the best result of the two attempts was recorded.

Running was assessed based on a 7 m shuttle run conducted in the following manner. A cone was placed at a distance of 7 m from the starting point, with an additional 2–3 m secured beyond the cone. Then, the participant started at the signal, ran around the cone, and returned to the starting point as quickly as possible. Finally, the time taken was recorded to the nearest 0.1 s.

Hopping was evaluated based on the distance one covered in eight consecutive hops. The dominant foot was determined beforehand, and the distance hopped from the take-off point to the heel of the final landing point was measured in centimeters. The best result of two attempts was recorded.

Static balance was measured using “static balance_closed eyes” conducted in the following manner. The participant’s dominant foot was determined. Then, the participant stood with feet apart, lifted one leg to 90 degrees, and closed their eyes at the start signal. The test ended when the supporting foot moved or the raised foot touched the ground. The duration of balancing was recorded to the nearest 0.1 s, and the best result of two attempts was recorded.

Dynamic balance was measured using a balance beam (10 cm wide, 30 cm high, and 3 m long) in the following manner. The participant stood on the balance beam on one foot with their arms extended out to the sides. At the start signal, they began walking heel-to-toe along the length of the balance beam, walking (running prohibited) as quickly as possible while maintaining a straight line. The total time taken to reach the other end of the beam was measured to the nearest 0.1 s. If the participant fell off the balance beam, the test started again at the starting point of the beam. If the participant fell again, the test continued from the point of the fall.

Overhand throwing was evaluated by having participants throw five tennis balls (Velcro balls) at a target set at a distance of 4 m for boys and 3 m for girls. Points were awarded based on how near the throw was to the target’s center. Two, one, and zero points were awarded if the ball landed within a radius of 30 cm, 30–90 cm, and more than 90 cm, respectively. The total score was determined by summing the points obtained from the five throws.

Kicking was assessed by having participants kick five size 4 soccer balls at a goal target positioned at a distance of 5 m for boys and 4 m for girls. The participant ran from the starting line (2 m behind the kick line) and kicked the ball. Points were awarded based on the proximity of the ball to the goal center. Two, one, and zero points were awarded if the ball landed inside a 90 cm × 60 cm goal, within a 120 cm × 90 cm zone marked with lines, and outside these zones (or hitting the goalpost). The total score was calculated by summing the points obtained from the five kicks.

#### 2.2.2. HRF

##### Muscular Strength

Muscular strength was measured using a grip strength dynamometer. Participants adjusted the dynamometer’s handle for a comfortable fit and assumed a standing position with their feet shoulder-width apart and arms relaxed at their sides. Upon receiving a starting signal, they squeezed the dynamometer with maximal force for two seconds. Each hand was tested twice, and the highest recorded value was documented. Measurements were recorded to the nearest 0.1 kg.

##### Cardiorespiratory Endurance

Cardiorespiratory endurance was measured using a progressive aerobic cardiovascular endurance running test. Cones were placed 15 m apart, and an additional 2–3 m of clear space was allocated at the turnaround points (the start and finish lines). At the start signal, the participant continuously ran back and forth between the cones, ensuring that both feet completely crossed the 15 m line before the next audio cue. Failure to cross the line in time resulted in a warning, and the test concluded after two warnings. The total number of completed laps was recorded.

##### Flexibility

Flexibility was measured with a seated trunk flexion test performed in the following manner. The participant sat with their legs extended straight and feet flat against the measurement device. With the fingertips of the overlapped hands touching the measurement device, the participant bent forward as far as possible without bouncing. This posture was held for two seconds, and the distance reached by the fingertips was measured to the nearest 0.1 cm. The best result of two attempts was recorded.

### 2.3. Data Analysis

A frequency analysis was performed to determine the characteristics of the participants. Then, a multiple regression analysis was conducted to explore the relationships between the variables. Statistical significance was set at α = 0.05. In addition, it was considered that when the VIF (variance inflation factor) exceeded 10, the problem of multicollinearity had arisen. All statistical analyses were performed using SPSS 29.0 software.

## 3. Results

In multiple regression analysis, the variance inflation factor (VIF) is a criterion for testing multicollinearity, which refers to high correlations among independent variables. A VIF value exceeding 4.0 is indicative of multicollinearity problems [37]. In this study, with all VIF values for the independent variables remaining below 4.0, multicollinearity was not an issue.

### 3.1. Impact of Locomotor Movement Skills on HRF

Table 2 presents the results of the multiple regression analysis of the impact of locomotor movement skills on muscular strength. The coefficient of determination (R^2^) was 0.096, indicating that 9.6% of the variance in muscular strength was explained by the variables of locomotor movement skills. The overall regression model was statistically significant (*F*(v) = 10.066, *p* < 0.05). Individual regression coefficients revealed that jumping (*β* = 1.66, *t* = 2.122, *p* < 0.05) and hopping (*β* = 0.178, *t* = 2.399, *p* < 0.05) positively affect muscular strength. However, running (*β* = 0.04, *t* = 0.67, *p* < 0.05) did not have a statistically significant effect.

Table 3 presents the results of the multiple regression analysis of the impact of locomotor movement skills on cardiorespiratory endurance. The coefficient of determination (R^2^) was 0.214, indicating that 21.4% of the variance in cardiorespiratory endurance was explained by the variables of locomotor movement skills. The overall regression model was statistically significant (*F*(3, 287) = 25.843, *p* < 0.05). Individual regression coefficients revealed that jumping (*β* = 0.128, *t* = 3.303, *p* < 0.05) and hopping (*β* = 0.012, *t* = 3.298, *p* < 0.05) positively affect cardiorespiratory endurance. However, running (*β* = −1.174, *t* = −1.0503, *p* < 0.05) did not have a statistically significant impact on cardiorespiratory endurance.

Table 4 presents the results of the multiple regression analysis of the impact of locomotor movement skills on flexibility. The coefficient of determination (R^2^) was 0.037, indicating that 3.7% of the variance in flexibility was explained by the variables of locomotor movement skills. The overall regression model was statistically significant (*F*(3, 287) = 3.608, *p* < 0.05). Individual regression coefficients revealed that jumping (*β* = 0.082, *t* = 3.235, *p* < 0.05) positively affects flexibility. However, running (*β* = 1.129, *t* = 1.556, *p* < 0.05) and hopping (*β* = −0.004, *t* = −1.846, *p* < 0.05) did not have a statistically significant impact.

### 3.2. Impact of Stability Skills on HRF

Table 5 presents the results of the multiple regression analysis of the impact of stability skills on muscular strength. The coefficient of determination (R^2^) was 0.029, indicating that 2.9% of the variance in muscular strength was explained by the variables of stability skills. The overall regression model was statistically significant (*F*(2, 288) = 4.268, *p* < 0.05). Individual regression coefficients revealed that static balance (*β* = 0.101, *t* = 2.913, *p* < 0.05) positively affects muscular strength. However, dynamic balance (*β* = 0.011, *t* = 0.534, *p* < 0.05) did not have a statistically significant impact on muscular strength.

Table 6 presents the results of the multiple regression analysis of the impact of stability skills on cardiorespiratory endurance. The coefficient of determination (R^2^) was 0.047, indicating that 4.7% of the variance in cardiorespiratory endurance was explained by the variables of stability skills. The overall regression model was statistically significant *F*(2, 288) = 7.055, *p* < 0.05). Individual regression coefficients revealed that static balance (*β* = 0.153, *t* = 2.796, *p* < 0.05) positively affects cardiorespiratory endurance. In contrast, dynamic balance (*β* = −0.073, *t* = −2.193, *p* < 0.05) had a statistically significant negative effect on cardiorespiratory endurance.

Table 7 presents the results of the multiple regression analysis of the impact of stability skills on flexibility. The coefficient of determination (R^2^) was 0.042, indicating that 4.2% of the variance in flexibility was explained by the variables of stability skills. The overall regression model was statistically significant (*F*(2, 288) = 6.198, *p* < 0.05). Individual regression coefficients revealed that static balance (*β* = 0.087, *t* = 2.711, *p* < 0.05) positively affects flexibility. However, dynamic balance (*β* = −0.038, *t* = -1.943, *p* < 0.05) did not have a statistically significant impact on flexibility.

### 3.3. Impact of Manipulative Skills on HRF

Table 8 presents the results of the multiple regression analysis of the impact of manipulative skills on muscular strength. The coefficient of determination (R^2^) was 0.049, indicating that 4.9% of the variance in muscular strength was explained by the variables of manipulative skills. The overall regression model was statistically significant (*F*(2, 288) = 7.434, *p* < 0.05). Individual regression coefficients revealed that overhand throwing (*β* = 0.538, *t* = 3.034, *p* < 0.05) positively affects muscular strength. However, kicking (*β* = 0.476, *t* = 1.936, *p* < 0.05) did not have a statistically significant impact on muscular strength.

Table 9 presents the results of the multiple regression analysis of the impact of manipulative skills on cardiorespiratory endurance. The coefficient of determination (R^2^) was 0.035, indicating that 3.5% of the variance in cardiorespiratory endurance was explained by the variables of manipulative skills. The overall regression model was statistically significant (*F*(2, 288) = 5.111, *p* < 0.05). Individual regression coefficients revealed that overhand throwing (*β* = 0.774, *t* = 2.721, *p* < 0.05) positively affects cardiorespiratory endurance. However, kicking (*β* = 0.508, *t* = 1.285, *p* < 0.05) did not have a statistically significant impact on cardiorespiratory endurance.

Table 10 presents the results of the multiple regression analysis of the impact of manipulative skills on flexibility. The coefficient of determination (R^2^) was 0.014, indicating that 1.4% of the variance in flexibility was explained by the variables of manipulative skills. The overall regression model was statistically significant (*F*(2, 288) = 2.089, *p* < 0.05). Individual regression coefficients revealed that overhand throwing (*β* = −0.346, *t* = −2.044, *p* < 0.05) negatively affects flexibility. However, kicking (*β* = 0.061, *t* = 0.258, *p* < 0.05) did not have a statistically significant impact on flexibility.

## 4. Discussion

This study investigated the relationship between FMSs and HRF among Korean first and second graders. By analyzing FMSs and HRF, this study aimed to establish foundational data for developing a physical education curriculum tailored to the motor development stages and fitness levels of lower elementary school students.

### 4.1. Interpretation of the Findings

#### 4.1.1. Impact of Locomotor Movement Skills on HRF

Locomotor movement skills improve cardiovascular health, promote muscle development, and reduce the risk of obesity. These benefits can further increase participation in physical activity, laying a strong foundation for lifelong physical fitness [38,39]. As shown in Table 2, Table 3 and Table 4, locomotor movement skills (jumping, running, and hopping) have a greater effect on cardiorespiratory endurance than on muscular strength and flexibility. The coefficient of determination (R^2^) for the muscular strength and flexibility models was 0.096 and 0.037, respectively, indicating low explanatory power. In contrast, the cardiorespiratory endurance model exhibited a higher R^2^ of 0.214, suggesting a stronger impact of locomotor movement skills on this aspect of HRF. This finding aligns with the findings of previous studies demonstrating a positive correlation between FMS scores and cardiorespiratory endurance [31,34,40]. Cardiorespiratory endurance has been found to predict reduced risk of cardiovascular diseases and premature death [41,42]. Therefore, incorporating FMSs into physical education programs for lower elementary school grades may help to prevent cardiovascular diseases in adulthood.

According to our analysis of individual locomotor movement skills, jumping positively influenced all HRF elements, particularly muscular strength and flexibility. This finding aligns with the findings of previous studies demonstrating that greater muscular strength translates to higher jump power, and increased flexibility enhances the range of motion, ultimately improving jumping ability [43,44]. Hopping positively affected muscular strength and cardiorespiratory endurance, but not flexibility. This finding aligns with the findings of previous studies indicating that greater muscular strength allows for higher jumps, while higher cardiorespiratory endurance aids in sustained exercise performance [45,46]. Running, however, showed no significant impact on muscular strength, flexibility, and cardiorespiratory endurance. This finding aligns with the findings of previous studies highlighting that running primarily utilizes fast-twitch muscle fibers optimized for short bursts of power, which leads to quicker fatigue [47]. These findings underscore the utility of running records in identifying sprinting talent at an early stage and the need to incorporate complementary cardiorespiratory endurance training for the development of well-rounded fitness.

Overall, the findings demonstrate that locomotor movement skills positively influence overall HRF among first and second graders, particularly cardiorespiratory endurance. Among the locomotor movement skills examined, jumping proficiency is the most significant contributor to HRF improvement.

#### 4.1.2. Impact of Stability Skills on HRF

Stability skills, which encompass the ability to maintain body balance and posture, are foundational skills for the effective performance of other motor skills [48]. As shown in Table 5, Table 6 and Table 7, the coefficient of determination (R^2^) for the muscular strength, cardiovascular endurance, and flexibility models was relatively low (0.029, 0.047, and 0.042, respectively), indicating limited explanatory power in the variability in HRF. However, all the models yielded statistically significant F-values. These findings suggest that stability skills influence HRF elements.

Static balance (measured using the single-leg stance test) positively influenced all HRF elements, suggesting a positive correlation between static balance performance and HRF. This result has been supported by the findings of previous studies demonstrating that muscular strength is crucial for maintaining static balance and preventing falls, high cardiorespiratory endurance enhances fatigue resistance, and flexibility increases the range of motion, ultimately improving the ability to balance [49,50,51]. In contrast, dynamic balance (measured using the balance beam walking test) did not affect muscular strength or flexibility but negatively influenced cardiorespiratory endurance. This could be due to the weak correlation between static and dynamic balance among children and the slower development of dynamic balance because it requires more complex neuromuscular function and practice [52,53]. Additionally, similar to running, negative correlation with cardiorespiratory endurance might be useful in identifying sports in which slow-twitch muscle fibers dominate.

Overall, the findings suggest that static balance skills, such as the ability to maintain balance while standing on one leg, may have a greater influence on overall HRF than dynamic balance skills, such as walking on a balance beam, among first and second graders.

#### 4.1.3. Impact of Manipulative Skills on HRF

Manipulative skills, which refer to the ability to handle objects, play an important role in the overall development of children and require coordination and precise motor control among various body parts [48]. As shown in Table 8, Table 9 and Table 10, the coefficient of determination for the muscular strength, cardiorespiratory endurance, and flexibility models was low (0.049, 0.035, and 0.014, respectively), indicating that the models did not fully explain the variability in HRF. However, statistically significant F-values were obtained, suggesting the potential influence of overhand throwing on overall HRF.

It is worth noting that overhand throwing, while positively impacting muscular strength and cardiorespiratory endurance, had a negative effect on flexibility. This finding aligns with the findings of previous studies showing that increased muscular strength enhances throwing speed and accuracy, and athletes with high cardiorespiratory endurance achieve a high level of throwing performance through continuous training [54,55]. However, higher flexibility might lead to less throwing accuracy due to reduced muscle stiffness, which can diminish the control of the arm, shoulder, and torso muscles necessary for the overhand throwing motion [56].

Kicking did not significantly affect any elements of HRF. Unlike overhand throwing, which primarily involves upper body and arm movements, kicking requires coordination between the lower and upper body, necessitating more complex neuromuscular adjustments that are typically attained at a later stage of development [48,57]. Therefore, the lack of maturity in kicking skills might make it difficult to find a direct connection with HRF.

### 4.2. Practical Implications of the Findings

The three types of FMSs (locomotor movement skills, stability skills, and manipulative skills) demonstrated varying influences on the elements of HRF. Among locomotor movement skills, jumping and hopping significantly impacted muscular strength and cardiorespiratory endurance. Of the two stability skills tested, static balance, measured by the single-leg stance test, significantly influenced all three HRF elements (muscular strength, cardiorespiratory endurance, and flexibility). Of the two manipulative skills tested, overhand throwing significantly influenced muscular strength and cardiorespiratory endurance. Overall, jumping had a particularly significant effect across all three elements of HRF. This suggests that incorporating activities emphasizing jumping in physical education programs for first and second graders could significantly contribute to promoting well-rounded HRF development.

Globally, a great proportion of elementary students fail to reach a mature level in FMSs [58,59]. This underscores the need for systematic and tailored exercise programs designed according to developmental stages from early childhood to adolescence [60]. Additionally, integrating FMSs and HRF in the physical education curriculum of lower elementary grades could facilitate a smooth transition to existing HRF programs for adolescents [33,35]. However, to overcome challenges stemming from the lack of knowledge of the concept and instruction of FMSs, it is essential to preemptively implement teacher training programs, develop evaluation tools, and establish continuous professional development programs [61].

Several limitations were found in the process of obtaining the results. Suggestions for future studies center on these limitations. First, most of the models tended to have low coefficients of determination, indicating that the regression models did not fully explain the variability in HRF. However, all models yielded statistically significant F-values. This suggests a potential influence of the included variables on HRF elements. Future research should explore this further by incorporating additional variables or using different measurement methods. Second, it is necessary to delve deeper into the impact of each skill on individual HRF elements and to strengthen the generalizability of the findings by expanding and diversifying the study population. Additionally, incorporating additional variables that might influence HRF is crucial for improving the model’s explanatory power. Finally, employing cross-validation techniques would enhance the model’s reliability.

## 5. Conclusions

Among first and second graders, the FMSs of jumping, hopping, static balance, and overhand throwing positively affect the HRF components of muscular strength, cardiorespiratory endurance, and flexibility. Physical education programs emphasizing these FMSs may promote overall HRF among younger elementary school children. Our results also show that jumping and maintaining static balance (as used, for example, in single-leg stance) have the strongest positive associations with the three elements of HRF. Prioritizing these skills in early elementary physical education may have a particularly significant impact on children’s healthy development. This study is expected to contribute to the development of physical education curricula for first and second graders by establishing the relationship between FMSs and HRF elements and determining the appropriate weighting of these elements in the curriculum.

## Figures and Tables

**Table 1:** Demographic characteristics of the participants.

Characteristic	Categories	Total	
		Frequency (n)	Percentage (%)
Sex	Boy	142	48.8
	Girl	149	51.2
Grade	First grade	137	47.1
	Second grade	154	52.9
Total		291	100.0

**Table 2:** Results of analyzing the impact of locomotor movement skills on muscular strength.

Independent Variables	Unstandardized Coefficients		Standardized Coefficients (*β*)	*t*	*p*	*VIF*
	B	Standard Error				
Jumping	0.055	0.026	1.66	2.122	0.035	1.921
Running	0.050	0.750	0.004	0.67	0.974	1.389
Hopping	0.006	0.002	0.178	2.399	0.017	1.743
Constant	1.912	6.135				
R^2^ = 0.096, *F* = 10.066, *p* < 0.05						

B: regression coefficient, *β* = beta, VIF: variance inflation factor, *F* = fault.

**Table 3:** Results of analyzing the impact of locomotor movement skills on cardiovascular endurance.

Independent Variable	Unstandardized Coefficients		Standardized Coefficients (*β*)	*t*	*p*	*VIF*
	B	Standard Error				
Jumping	0.128	0.039	0.240	3.303	0.001	1.921
Running	−1.174	1.114	−0.065	−1.053	0.293	1.389
Hopping	0.012	0.004	0.229	3.298	0.001	1.743
Constant	0.472	9.113				
R^2^ = 0.214, F = 25.843, *p* < 0.05						

**Table 4:** Results of analyzing the impact of locomotor movement skills on flexibility.

Independent Variables	Unstandardized Coefficients		Standardized Coefficients (*β*)	*t*	*p*	*VIF*
	B	Standard Error				
Jumping	0.082	0.025	0.261	3.235	0.001	1.921
Running	1.129	0.726	0.107	1.556	0.121	1.389
Hopping	−0.004	0.002	−0.142	−1.846	0.066	1.743
Constant	−4.270	5.937				
R^2^ = 0.037, *F* = 3.608, *p* < 0.05						

**Table 5:** Results of analyzing the impact of stability skills on muscular strength.

Independent Variables	Unstandardized Coefficients		Standardized Coefficients (*β*)	*t*	*p*	*VIF*
	B	Standard Error				
Static balance	0.101	0.035	0.171	2.913	0.004	1.012
Dynamic balance	0.011	0.021	0.031	0.534	0.594	1.012
Constant	11.115	0.934				
R^2^ = 0.029, *F* = 4.268, *p* < 0.05						

**Table 6:** Results of analyzing the impact of stability skills on cardiovascular endurance.

Independent Variables	Unstandardized Coefficients		Standardized Coefficients (*β*)	*t*	*p*	*VIF*
	B	Standard Error				
Static balance	0.153	0.055	0.162	2.796	0.006	1.012
Dynamic balance	−0.073	0.033	−0.127	−2.193	0.029	1.012
Constant	17.398	1.474				
R^2^ = 0.047, *F* = 7.055, *p* < 0.05						

**Table 7:** Results of analyzing the impact of stability skills on flexibility.

Independent Variables	Unstandardized Coefficients		Standardized Coefficients (*β*)	*t*	*p*	*VIF*
	B	Standard Error				
Static balance	0.087	0.032	0.158	2.711	0.007	1.012
Dynamic balance	−0.038	0.020	−0.113	−1.943	0.053	1.012
Constant	8.984	0.870				
R^2^ = 0.042, *F* = 6.198, *p* < 0.05						

**Table 8:** Results of analyzing the impact of manipulative skills on muscular strength.

Independent Variables	Unstandardized Coefficients		Standardized Coefficients (*β*)	*t*	*p*	*VIF*
	B	Standard Error				
Overhand throwing	0.538	0.177	0.177	3.034	0.003	1.020
Kicking	0.476	0.246	0.113	1.936	0.054	1.020
Constant	10.444	0.748				
R^2^ = 0.049, *F* = 7.434, *p* < 0.05						

**Table 9:** Results of analyzing the impact of manipulative skills on cardiovascular endurance.

Independent Variables	Unstandardized Coefficients		Standardized Coefficients (*β*)	*t*	*p*	*VIF*
	B	Standard Error				
Overhand throwing	0.774	0.285	0.160	2.721	0.007	1.020
Kicking	0.508	0.395	0.075	1.285	0.200	1.020
Constant	14.078	1.201				
R^2^ = 0.035, *F* = 5.111, *p* < 0.05						

**Table 10:** Results of analyzing the impact of manipulative skills on flexibility.

Independent Variables	Unstandardized Coefficients		Standardized Coefficients (*β*)	*T*	*p*	*VIF*
	B	Standard Error				
Overhand throwing	−0.346	0.169	−0.121	−2.044	0.042	1.020
Kicking	0.061	0.235	0.015	0.258	0.797	1.020
Constant	9.655	0.714				
R^2^ = 0.014, *F* = 2.089, *p* < 0.05						

## Data Availability

The data presented in this study are available upon request from the corresponding author. The data are not publicly available because of the protection of personal information.
